# Neurofeedback training for alcohol dependence versus treatment as usual: study protocol for a randomized controlled trial

**DOI:** 10.1186/s13063-016-1607-7

**Published:** 2016-10-03

**Authors:** W. Miles Cox, Leena Subramanian, David E. J. Linden, Michael Lührs, Rachel McNamara, Rebecca Playle, Kerenza Hood, Gareth Watson, Joseph R. Whittaker, Raman Sakhuja, Niklas Ihssen

**Affiliations:** 1 Cardiff University School of Medicine, Hadyn Ellis Building, Maindy Road, Cathays, Cardiff, CF24 4HQ UK; 2 School of Psychology, Tower Building, Cardiff University, 70 Park Place, Cardiff, CF10 3AT UK; 3 Department of Cognitive Neuroscience, Maastricht University, Oxfordlaan 55, 6229 Maastricht, The Netherlands; 4 South East Wales Trials Unit (SEWTU), Centre for Trials Research, Cardiff University, College of Biomedical and Life Sciences, Neuadd Meirionnydd, Heath Park, Cardiff, CF14 4XN UK; 5 Cwm Taf University Health Board, Llwyn yr Eos Clinic, Main Road, Church Village, Cardiff, CF38 1RN UK; 6 Department of Psychology, Wolfson Building, Durham University, Queen’s Campus, Stockton-on-Tees, TS17 6BH UK

**Keywords:** Alcohol dependence, Neurofeedback training, Functional magnetic resonance imaging, Randomised controlled trial, Relapse prevention

## Abstract

**Background:**

Real-time functional magnetic resonance imaging (rtfMRI) is used for neurofeedback training (NFT). Preliminary results suggest that it can help patients to control their symptoms. This study uses rtfMRI NFT for relapse prevention in alcohol dependence.

**Methods/design:**

Participants are alcohol-dependent patients who have completed a detoxification programme within the past 6 months and have remained abstinent. Potential participants are screened for eligibility, and those who are eligible are randomly assigned to the treatment group (receiving rtfMRI NFT in addition to treatment as usual) or the control group (receiving only treatment as usual). Participants in both groups are administered baseline assessments to measure their alcohol consumption and severity of dependence and a variety of psychological and behavioural characteristics that are hypothesised to predict success with rtfMRI NFT. During the following 4 months, experimental participants are given six NFT sessions, and before and after each session various alcohol-related measures are taken. Participants in the control group are given the same measures to coincide with their timing in the experimental group. Eight and 12 months after the baseline assessment, both groups are followed up with a battery of measures. The primary research questions are whether NFT can be used to teach participants to down-regulate their brain activation in the presence of alcohol stimuli or to up-regulate their brain activation in response to pictures related to healthy goal pursuits, and, if so, whether this translates into reductions in alcohol consumption. The primary outcome measures will be those derived from the functional brain imaging data. We are interested in *improvements* (i.e., reductions) in participants’ alcohol consumption from pretreatment levels, as indicated by three continuous variables, not simply whether or not the person has remained abstinent. The indices of interest are *percentage of days abstinent*, *drinks per drinking day*, and *percentage of days of heavy drinking*. General linear models will be used to compare the NFT group and the control group on these measures.

**Discussion:**

Relapse in alcohol dependence is a recurring problem, and the present evaluation of the role of rtfMRI in its treatment holds promise for identifying a way to prevent relapse.

**Trial registration:**

ClinicalTrials.gov Identifier: NCT02486900, registered on 26 June 2015.

**Electronic supplementary material:**

The online version of this article (doi:10.1186/s13063-016-1607-7) contains supplementary material, which is available to authorized users.

## Background

There has been a longstanding interest in the use of biofeedback in the treatment of a variety of medical and psychiatric disorders. The basic principle of biofeedback is that if patients are provided with feedback about the normally involuntary and uncontrollable physiological responses associated with their illness, they can use mental strategies to control these responses and thereby improve their symptoms [1].

During the 1970s, there was a surge of interest in biofeedback using electroencephalography (EEG) Clinical syndromes for which it was attempted included tension headache, hypertension, chronic anxiety, and eating disorders. Although promising results were obtained, certain methodological problems prevailed, especially small sample sizes and the absence of satisfactory control conditions [2]. Thus, definitive conclusions about the efficacy of biofeedback could not be reached. Nevertheless, the interest in it prevailed into the 1980s and beyond, and at this time there was an increase in the number of ailments for which it was used. Additionally, EEG biofeedback was extended to cognitive enhancement and skill training for musicians and dancers and even surgeons, again with promising results. Today various kinds of biofeedback are being offered in certain countries for a variety of clinical problems, including addiction, anxiety, attention deficit/hyperactivity disorder (ADHD), depression, epilepsy, asthma, and chronic pain [3].

In the past decade, there has been a revival of scientific interest in biofeedback and its clinical applications, partly driven by the development of real-time functional magnetic resonance imaging (rtfMRI) neurofeedback training (NFT). For NFT, rtfMRI has advantages over EEG. For example, it localises brain signals to specific areas of the brain with more precise resolution, and it capitalises on the fact that relevant brain areas can be activated when patients only imagine particular events happening (e.g. moving a limb of the body, having a drink of alcohol). rtfMRI NFT has already been used in pilot studies to help patients with a variety of disorders to ameliorate their symptoms or unwanted behaviour. These include patients with debilitating pain [4], Parkinson’s disease [5], depression [6], and nicotine addiction, or alcohol [7–10]. The feedback that patients receive about their brain reactions and their ability to regulate them seems to have an added benefit in that it helps them to acquire a sense of control over their symptoms (e.g. [8]). Despite these promising initial results, additional research is needed to identify (1) variables that determine the effectiveness of rtfMRI NFT, and (2) optimal strategies for using it in clinical applications.

### BRAINTRAIN: taking imaging into the therapeutic domain

BRAINTRAIN (http://www.braintrainproject.eu/) is a research project supported by the European Commission under the Health Cooperation Work Programme of the 7th Framework Programme that is seeking to achieve these goals. BRAINTRAIN, coordinated by coauthor DL at Cardiff University, is a 4-year project to (1) further test whether rtfMRI is an effective additional form of treatment for people suffering from a variety of mental and behavioural disorders, and (2) identify variables that determine the effectiveness of rtfMRI NFT. The disorders that are being investigated include autism spectrum disorder (Universidade de Coimbra, Portugal), alcohol dependence (Cardiff University), posttraumatic stress disorder (Tel Aviv University, Israel), binge-eating disorder (Eberhard Karls Universität Tübingen, Germany), and childhood anxiety (University of Oxford/King’s College London).

The purpose of the present article is to describe the component of this research programme that is being conducted at Cardiff University. It is an early phase randomised feasibility trial to assess the effectiveness of rtfMRI NFT in the treatment of alcohol dependence.

### Study objectives

The main aims of the Cardiff arm of BRAINTRAIN are (1) to determine whether participants who receive rtfMRI NFT can down-regulate their brain activation while they are exposed to alcohol stimuli (or up-regulate their brain activation in response to pictures related to healthy goal pursuits), and (2) to determine whether, compared to control participants, the training helps patients to reduce their urges to drink and their consumption of alcohol and to bring about other improvements in their functioning. Additionally, secondary aims of the trial are to (1) identify participant characteristics that are related to success with the training, and (2) refine the parameters of the training in order to achieve an optimal intervention.

## Methods

### Ethical, safety, and privacy issues

The Wales Research Ethics Committee 1 approved the research protocol (Ref: 14/WA/1172). Additionally, the Research and Development Committee at Cwm Taf University Health Board, Cardiff and Vale University Health Board, and Aneurin Bevan University Health Board approved the protocol. Plans for modifying the trial protocol are unforeseen; however, should they arise, approval will be sought from the relevant ethics committees. Cardiff University as sponsor of the trial reserves the right to audit trial conduct should they have any concerns regarding conduct or safety. The South East Wales Trials Unit (SEWTU) is providing oversight of trial conduct. The protocol was designed in accordance with the SPIRIT (Standard Protocol Items: Recommendations for Interventional Trials) guidelines for interventional trials (Additional File 1).

A magnetic resonance imaging (MRI) safety-screening questionnaire is used to screen potential participants for MRI scanning. Patients who have contraindications for MRI scanning, such as metal implants in their bodies, are excluded. All information and data obtained from participants during the course of the study will be kept confidential by the research team in accordance with the UK Data Protection Act 1998.

### Design

**Fig. 1:**
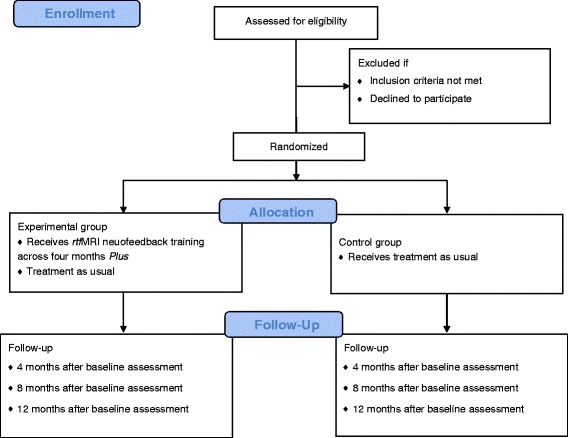
Consolidated Standards of Reporting Trials (CONSORT) flow diagram of the phases of the BRAINTRAIN trial

The study is an unblinded, early-phase randomised feasibility trial that includes an experimental group and a control group of alcohol-dependent participants. The experimental group (*n* = 25) receive *f*MRI NFT in addition to treatment as usual (TAU). The control group (*n* = 25) receive only TAU. Randomisation is balanced for time since detoxification (more than 3 months, less than 6 months). The South East Wales Trials Unit is providing an online randomisation programme. A flowchart showing the sequence of recruitment, assessment, and intervention is shown in Fig. 1. A Standard Protocol Items: Recommendations for Interventional Trials (SPIRIT) diagram of the study period is shown in Table 1.

**Table 1:** Standard Protocol Items: Recommendations for Interventional Trials (SPIRIT) diagram

	Study period					
	Enrolment	Allocation				
Timepoint	Pre intervention	Time 0	Baseline	Post intervention	8-month follow-up	12-month follow-up
Enrolment:						
Eligibility screen	X					
Informed consent	X					
Allocation		X				
Interventions:						
rtfMRI NFT + TAU				X		
TAU				X		
Assessments:						
Alcohol Timeline Followback	X		X	X	X	X
Drinking Urges Questionnaire			X	X	X	X
Obsessive Compulsive Drinking Scale			X	X	X	X
Alcohol Stroop Test			X	X	X	X
Severity of Alcohol Dependence			X			
Thought Control Questionnaire			X			
Thought Control Ability Questionnaire			X			
Profile of Mood States			X		X	X
Hospital Anxiety and Depression Scale			X	X	X	X
Beck Depression Inventory			X	X	X	X

*rtfMRI* real-time functional magnetic resonance imaging, *TAU* treatment as usual

### Participant eligibility, recruitment, and screening

Eligible participants are alcohol-dependent patients who have successfully completed an inpatient detoxification programme 1 to 6 months before enrolment and are currently enrolled as outpatients. The programmes through which patients have been detoxified include those run by the Cwm Taf University Health Board, Cardiff and Vale University Health Board, and Aneurin Bevan University Health Board. Additional recruitment sites might be added at a later time. The TAU that patients receive includes psychological support, psychoeducation, and medical management of abstinence, for example with anticraving medication or disulfiram. Participants are randomly assigned to (1) a control group who continue to receive only TAU, or (2) an experimental group who receive rtfMRI NFT in addition to TAU.

Potential participants’ diagnosis of alcohol dependence (*International Classification of Diseases*, *tenth edition* (ICD-10): F102) is confirmed by inspection of their clinical records. Patients meeting this criterion are initially identified and approached to judge their interest in and suitability for participating in the study. These contacts are made either by (1) clinicians who are directly involved in the care of the patients, or (2) staff from the Health and Care Research Workforce in Wales.

Patients who are identified as suitable for the study are either invited personally by their primary clinician or they are sent a letter of invitation by their clinical team. Patients are also offered the opportunity to obtain more information about the study and to ask questions at informational events called *open days*, which are organised at the recruitment sites. During the open days, one or more members of the research team present details about the procedures and practicalities of the study (e.g. scanning procedures, questionnaires that are being used).

Once suitable patients have indicated an interest in participating in the study, they are invited to visit either one of the community alcohol and drug clinics or our research laboratory at Cardiff University. In either case, a research officer will give patients an information sheet about the study procedure, and will also ask participants to provide written informed consent to participate in the study (see Additional Files 2 and 3). If consent is obtained, participants are asked to complete several screening assessments as follows:

1. The Mini International Neuropsychiatric Interview (MINI; [11]) is a short, structured interview for diagnosing psychiatric disorders based on *Diagnostic and Statistical Manual of Mental Disorders, fourth edition* (DSM-IV) and ICD criteria. Only the psychosis section of the MINI is administered. Patients identified as having a history of a psychotic disorder not related to alcohol dependence are excluded from participating
2. The Wechsler Abbreviated Intelligence Scale (WASI-II; [12]) provides a global measure of patients’ level of intellectual functioning. Participants with an IQ below 70 are excluded because they would likely find the experimental tasks too difficult to complete
3. An in-house drug-use questionnaire measures participants’ use of illicit substances. Patients who have on-going regular use of illicit drugs, except for cannabis, are excluded
4. The Alcohol Timeline Followback (TLFB) Questionnaire [13] is used to determine whether patients have drunk alcohol since their discharge from their detoxification programme. Patients who have drunk any alcohol are excluded

Participants are informed that they are free to withdraw from the study at any time.

### Baseline assessment

During the baseline assessment, participants complete the following instruments: a demographic questionnaire (which asks about participants’ age, gender, level of education, and socioeconomic status); a National Health Service (NHS) resources utilisation questionnaire (which asks about the use of prescription medications and treatment services); and various standardised measures which are shown in Table 2. Patients are randomised after the baseline session.

**Table 2:** Standardised measures administered

Measure	Description
Alcohol Timeline Followback (TLFB) Questionnaire [13]; primary outcome measure	Measures alcohol use during the 4 months immediately prior to patients’ entering treatment
Drinking Urges Questionnaire [22]; secondary outcome measure	Assesses participants’ desire to drink, expectations of positive effects following drinking, relief of withdrawal and negative affect following drinking, and intentions to drink
Obsessive Compulsive Drinking Scale (OCDS) [23]; secondary outcome measure	Measures respondents’ obsessive thoughts about alcohol use and compulsive behaviours related to drinking
Alcohol Stroop Test [24]; secondary outcome measure	Measures alcohol-related attentional distraction by alcohol-related stimuli
Severity of Alcohol Dependence Questionnaire [25]; primary outcome measure	Measures the degree to which patients are physically and psychologically dependent on alcohol
Thought Control Questionnaire (TCQ) [26]; BRAINTRAIN core outcome measure	Assesses the effectiveness of strategies used to control unpleasant or unwanted thoughts
Thought Control Ability Questionnaire (TCAQ) [27]; BRAINTRAIN core outcome measure	Measures individuals’ perceived ability to control unwanted and intrusive thoughts
Profile of Mood States (POMS) [28]; BRAINTRAIN core outcome measure	Measures anger, confusion, depression, fatigue, tension, and vigour
Hospital Anxiety and Depression Scale (HADS) [29]; BRAINTRAIN core outcome measure	Measure anxiety and depression
Beck Depression Inventory (BDI) [30]; BRAINTRAIN core outcome measure	Measures depression

### Assessment at NFT sessions

At each of the six NFT sessions across 4 months, patients in the experimental group complete the Drinking Urges Questionnaire (before and after the MRI scan), a self-rating of craving during the scanning session, the Alcohol TLFB (only after the last training session and covering the period from the baseline assessment to the present), the Alcohol Stroop Test (only after the last training session); the OCDS (only after the last training session); the HADS (only at the last training session); the BDI (only at the last training session); a debriefing interview questionnaire to identify strategies that participants used to down-regulate their brain reactions to alcohol stimuli (and up-regulate their brain responses to other goal-related pictures), their general experience with the procedure, and any adverse reactions that they might have experienced.

### Four-month assessment of the control group

Four months after their baseline assessment, patients in the control group complete the Alcohol TLFB (covering the period from the baseline assessment to the present), the NHS resource utilisation questionnaire, the Drinking Urges Questionnaire, the Alcohol Stroop Test, the OCDS, and the POMS.

### Follow-up assessments

Eight and 12 months after the baseline assessment, both the experimental group and the control group are administered the Alcohol TLFB (covering the period from the previous assessment to the present), the NHS resource utilisation questionnaire, the Drinking Urges Questionnaire, the Alcohol Stroop Test, the OCDS, the POMS, the HADS, and the BDI.

### BRAINTRAIN core outcome measures

Some of the assessment instruments named above are part of the BRAINTRAIN core outcome measures, i.e. they are given in all five of the centres involved in the BRAINTRAIN project. Some of them (e.g. those measuring intellectual functioning and thought control) are used mainly to predict who will perform better at NFT and benefit more from it clinically. These measures will be used to stratify patients, and they are largely exploratory. Other instruments in this set measure nonspecific clinical factors and comorbidities (e.g. depression, anxiety) and are included across the different trials to increase the power of secondary outcome measures and to examine nonspecific effects of NFT on mental health (across different diagnoses and research protocols).

Participants are being sent reminders to attend the follow-up sessions.

Whenever possible, outcome data will be collected for all participants whether or not they adhere to the planned intervention. The data will be analysed according to the intention-to-treat principle.

### Intervention

During the baseline assessment, participants also perform a computer-based rating task, in which they are presented with a total of 100 pictures showing different categories of alcoholic beverages (wine, beer, spirits, etc.) and pub scenes and 100 pictures illustrating alternative, healthy goals (positive personal relationships, health and fitness, employment and career, further education/self-improvement, and personal finances). For each alcohol-related picture, participants are asked to indicate (by using the mouse to move the cursor along a visual analogue scale on the screen) how much this specific picture makes them want a drink of alcohol; response options range from *not at all* to *very strongly*. Using the same response modalities, participants rate each of the pictures related to an alternative goal with regard to how much it reminds them of a positive goal that they are currently pursuing. For each of the subsequent NFT sessions in which alcohol-related pictures are down-regulated or alternative goal-related pictures are up-regulated, the pictures are randomly selected from the 14 pictures in each category that the participant rated most highly. Thus, the stimulus sets are tailored to each participant’s specific drinking preferences and hierarchy of alternative goals.

The NFT involves magnetic resonance imaging (MRI) during which patients lie on a movable bed inside the bore of an MRI scanner. Before entering the MRI suite, patients undergo a detailed MRI safety screening (as described earlier), including a determination of potential safety hazards (see exclusion criteria) and the removal of all metal objects, such as keys or jewellery, from their body.

While in the scanner, participants in the experimental group are again exposed to alcohol-related stimuli, such as pictures of alcoholic drinks, and they are trained to regulate their responses in specific brain regions that are activated by the stimuli. In addition, participants are exposed to pictures showing alternative, socially desirable goals, such as family/relationships or employment. The rationale for including the two categories of stimuli is that we earlier demonstrated at a neural level that heavy drinking is associated with overvaluation of alcohol and undervaluation of alternative, socially desirable goals [14]. Moreover, consistent with motivational theory [15–17], it has been shown that problem drinkers’ success in reducing their drinking is associated with the degree to which they have other satisfying alternative goals to pursue and enjoy [16].

The size of the images that participants see corresponds to the level of activation. They are told to use any kind of mental strategy at their disposal (e.g. thinking about the negative consequences of drinking) that helps to reduce size of the image (in the case of alcohol pictures) or increase size of the image and thereby the degree of brain activation (in the case of healthy, goal-related pictures).

Experimental participants have six NFT training sessions spread across 4 months, and they are also asked to practice at home the mental strategies they used in the MRI scanner. They also (1) rate their urges to drink before, during, and after each training session, and (2) complete questionnaires and other tests to measure their alcohol consumption and other relevant aspects of their behaviour. Control participants complete the same measures that are timed to coincide with their administration to the experimental group. The primary outcome measures are obtained 4 months after the baseline assessment. Eight and 12 months after baseline assessment both groups complete a follow-up assessment battery.

For the duration of the scanning session, several additional physiological parameters are measured, including heart rate via a finger pulse sensor and respiratory rate via a belt affixed to the participant’s chest. Participants are able to see outside the scanner during the scan, and radiographers are able to see and monitor participants from the control room. Participants are given a call button to press if they need to communicate with someone outside the scanner. The scan can be stopped at any time at a participant’s request (e.g. if he or she feels uncomfortable) or if the radiographer becomes concerned about a participant’s wellbeing.

Imaging data are acquired using the 3T General Electric HDx or Siemens Prisma scanners at the Cardiff University Brain Research Imaging Centre (CUBRIC) and standard parameters for functional and structural brain imaging. Blood oxygenation level-dependent (BOLD) signals during localiser and neurofeedback runs (see description below) are measured with a T2*-weighted gradient echo planar imaging (EPI) sequence that is synchronised to the onset of the stimulus presentation. Each functional EPI volume contains 24 slices of 2.5-mm thickness, with 0.5-mm inter-slice spacing (in-plane resolution = 3 mm, matrix size = 64 × 64, FoV (field of view) = 192 mm, TR (repetition time) = 1500 ms, TE (echo time) = 30 ms, flip angle = 80°, orientation = transversal). High-resolution structural images are acquired before the first functional scan using a fast spoiled gradient echo sequence (FSPGR) with 172 contiguous sagittal slices of 1-mm thickness (voxel size: 1 × 1 × 1 mm, TR = 7.9 s, TE = 3.0 ms, flip angle = 20°, FoV = 256 × 256 × 172 mm).

**Fig. 2:**
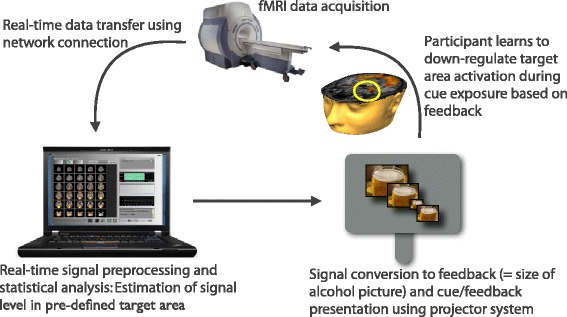
Illustration of the neurofeedback setup used in the intervention group. Participants receive real-time feedback about the computed activation level in target areas that have been identified in a preceding *localiser* scan as key components of the motivational network involved in alcohol-cue reactivity. Activation levels are represented as varying picture sizes, with decreasing picture sizes reflecting successful down-regulation

The basic neurofeedback setup being used in the trial is illustrated in Fig. 2. Patients are asked to lie still throughout the scanning sessions; they are in the scanner for no longer than 90 min. During the scan, patients are exposed to pictorial stimuli (depicting either alcoholic drinks or alternative goals), which are projected onto a screen behind the scanner and viewed through a mirror attached on the MRI head coil. Functional MRI (*f*MRI) data are acquired in short blocks (*runs*) having a duration of 7.5 min. The first run serves as the localiser procedure in which we present in alternating blocks of 30-s alcohol-related pictures or goal-related pictures and neutral pictures (household objects). Each block contains 15 different pictures of the same category (2 s per picture) and is followed by a fixation (rest) period. Participants are instructed to attentively watch the pictures without any cognitive/emotional regulation of the responses that are elicited by these pictures. Based on real-time preprocessing and statistical analysis of the BOLD signal recorded during the localiser, a specific target brain area (either alcohol- or goal-related) is individually selected for each participant in each session. Statistically significant regions of interest (ROIs) are selected using a t-contrast of alcohol/goal-related versus neutral images, and a variable t-threshold to ensure that a robust cluster is activated in a functionally relevant area (minimum of four connected voxels).

The next four runs consist of two NFT runs, during which the participant attempts to self-regulate activation in the target area, and two mirror (M) runs in the order NFT-M-NFT-M. During mirror runs, the previous feedback-modulated stimulus is presented, but participants are instructed to passively view it and not attempt to regulate their response. These runs serve as a control for potential low-level image size effects on activation in the target area. They will allow a comparison to be made between the same stimulus presentation with and without attempted self-regulation. Selection of both sets of pictures is based on the computer-based rating task that the participant performed during the baseline session.

Each neurofeedback run consists of seven regulation blocks of 30-s duration showing one picture exemplar whose size changes according to the participant’s BOLD activation in the target area. Between regulation blocks, a 30-s resting block showing a fixation cross is interspersed, and each run begins with a 30-s rest block. Feedback is provided by varying the size of the picture, which can range from between 100 % of the entire projector screen (1024 × 768 pixels) and 10 % of the screen, depending on the level of activation.

Down-regulation is signalled by decreasing the size of the picture, following a procedure piloted by our research group for food-related stimuli [18]. Up-regulation (during exposure to other goal-related pictures) is signalled by increases in the size of the picture. The aspect ratio of the pictures is kept constant for all picture sizes. The size of the picture is changed based on the percentage of the signal change in the current block compared to the baseline in the previously defined ROI. The last five volumes prior to the current neurofeedback block are used to calculate the baseline level.

A moving average filtre of four volumes is used to overcome spikes and signal drops and to avoid large fluctuations in the size of the pictures. The percentage signal change is calculated using this equation:

$$ fb = \left( val-bl\right)/bl\ast 100 $$

The percentage signal change (PSC) is then normalised to a value between −1 and +1 by dividing the *fb* value by a predefined maximum PSC of 1 %. This is done in order to be able to resize the pictures in the predefined way.

Between the *f*MRI runs, the experimenter speaks to the participant using an intercom and gives the participant the opportunity to rest. Participants in the control group are invited to attend four behavioural assessment sessions (baseline assessment, primary assessment after 4 months, and a follow-up assessment after 8 and 12 months).

All adverse events and serious adverse events (SAEs) will be recorded on the appropriate Case Report Form. Events meeting the criteria for a SAE will be reported according to the timelines specified in the study protocol (e.g. an SAE form should be completed for all SAEs within 24 h).

Post trial, all participants will remain under the care of their primary treating physician. Cardiff University will provide indemnity and compensation in the event of a claim by a participant, or by someone on a participant’s behalf, for negligent harm as a result of the study design and/or in respect of the protocol authors/research team. Cardiff University does not provide compensation for non-negligent harm.

### Sample size estimation

There are no published randomised trials to inform a sample size calculation for the early phase efficacy trial of the neurofeedback intervention that is being evaluated. However, experimental neurofeedback studies have found large effect sizes of 0.5 to 1.5, depending on the patient group that was tested [5, 6]. At a significance level of 0.05, the present sample size (from which we expect a 20 % dropout rate, yielding a final size of 40 (or 20 in each group)) will allow us to detect effect sizes ranging from 0.8 with 70 % power to 1.1 with 90 % power. This level is suitable for single-site, early-phase detection of clinically promising results.

### Analyses

One of the study investigators will perform data entry, and a second member of staff will compare the database record (the electronic Case Report Form; eCRF) with the source record (the paper Case Report Form; pCRF). pCRFs will be stored securely in locked filing cabinets, and the data in the eCRFs will be stored securely in the database housed at Cardiff University. Access to the database is restricted to named study team personnel only and in accordance with their specific roles and responsibilities. The data management plan will contain all relevant details about how the data for the study are being handled and managed.

The study site statistician, the principal investigator (PI), and the trial unit statistician will have access to the final dataset for study site-specific analyses and meta-analyses across the sites in BRAINTRAIN. The analysis population will be on an intention-to-treat basis (as randomised) using complete data. We do not intend to perform imputation for missing data in this early phase study but will check for bias in any missing cohort.

The anatomical and functional brain imaging data will be analysed using BrainVoyager (http://www.brainvoyager.com) or another appropriate software package. A general linear model will be used to test the effects of NFT on brain activation, both in target areas and at the whole-brain level. Specifically, we will contrast activation in neurofeedback runs with passive viewing during the perceptual control (mirror) runs. We expect to find reduced activation during neurofeedback in the target and other craving-related areas, but increased activation in other areas, e.g. those related to emotional control or which represent alternative, socially desirable goals (in the runs that include the goal-related pictures). Participants’ improvement in self-regulation across the sessions will be analysed using brain-activation parameters in repeated the correct term is definitely measures (with an "s") NOT measure. Analyses of variance (ANOVAS). The ability to self-regulate will also be correlated with online and offline craving scores obtained during each session.

The behavioural data will be analysed using SPSS or another appropriate statistical software package. The primary analyses will compare the experimental and control groups on three drinking indices (percentage of days abstinent, drinks per drinking day, and percentage of days of heavy drinking) at the primary endpoint (4 months after the baseline assessment). For these analyses, a general linear model will be used. Covariates will include potential variables that determine participants’ success with the NFT (e.g. their age and gender and drinking indices at baseline) and the variables that were used to balance the two groups during randomisation. Effect sizes for the intervention will be evaluated using confidence intervals around the group difference. Analyses will be conducted separately for the follow-up assessments, again using general linear models adjusted for baseline covariates and randomisation variables. Outcome measures related to craving will be evaluated for both immediate and sustained effects of the NFT.

In exploratory analyses, data from the homework diaries will be examined for a potential relationship between the amount of self-regulation practice that participants report and their scores on craving and alcohol consumption. Finally, the relationship between nonalcohol-related measures (e.g. thought control ability, intellectual ability) and the alcohol-related outcome measures will be examined using analysis of covariance (ANCOVA) or correlational and regression analyses. Nonspecific effects of the NFT on participants’ mood will be examined using the measures related to emotional processes (e.g. the POMS).

A publication policy will be developed and reviewed during the course of the trial, which will include details of dissemination plans to health care professionals. Publications from the BRAINTRAIN consortium will be coordinated by the Executive Committee of BRAINTRAIN (chaired by the PI) in collaboration with the funding organisation. The trial results will be submitted for publication in an online open access journal.

## Discussion

Alcohol dependence is a recalcitrant disorder, with high rates of relapse to heavy drinking occurring following treatment [19, 20]. In recent years, new medications and psychosocial interventions have been tested, but with disappointing effects on rates of relapse [19]. In view of the encouraging results obtained with rtfMRI NFT in the case of other psychiatric and behavioural disorders [4–9], its use in the treatment of alcohol dependence offers great promise.

The present study allows us to evaluate the overall effectiveness of rtfMRI NFT in reducing patients’ urges to drink and various indices of their alcohol consumption. Additionally, we will be able to evaluate participant characteristics (e.g. the ability to control thoughts, anxiety, depression, intellectual ability) that help to determine patients’ degree of success with the intervention. If positive results are obtained, more cost-effective techniques for helping patients to achieve self-control over their alcohol consumption could be developed and evaluated. Moreover, the use of these techniques could be extended to include early stage problem drinkers. Early stage problem drinkers are far more numerous in society than are dependent drinkers, yet dependent drinkers are far more costly to treat [21]. Thus, it could be highly effective and cost-effective to successfully intervene with people who are developing alcohol problems before these problems escalate into dependent drinking.

## Trial status

This trial is ongoing and participants are being actively recruited.

## Additional files

Additional file 1: SPIRIT checklist. (DOC 124 kb) Additional file 2: Consent Form (both groups). (DOCX 403 kb) Additional file 3: Consent Form (MRI training). (DOCX 132 kb)

## Abbreviation

rtfMRI: Real-time functional magnetic resonance imaging
