# Supplementary material for: Neurofeedback training for alcohol dependence versus treatment as usual: study protocol for a randomized controlled trial
Source: Trials. 2016 Oct 3;17:480. doi: 10.1186/s13063-016-1607-7 (PMC5048603; doi:10.1186/s13063-016-1607-7)
Supplement: Additional file 2: — Consent Form (both groups). (DOCX 403 kb) [file 13063_2016_1607_MOESM2_ESM.docx]

**Consent Form:** Neurofeedback & Alcohol Dependence

**Study part (group):** Baseline assessment/screening (both groups)

Prof David Linden, Dr Niklas Ihssen, Dr Leena Subramanian

Version 2, 14 November 2014

**CONSENT TO PARTICIPATE IN A RESEARCH STUDY**

Study Number:

Patient Identification Number:

**Title of Project:** Real-time fMRI neurofeedback as a treatment tool for alcohol dependence

**Name of Researchers:** Prof David Linden, Dr Niklas Ihssen, Dr Leena Subramaninan

Please initial box.

1. I confirm that I have read and understood the information sheet dated........................

(version ....) for the above study. I have had the opportunity to consider the

information and to ask questions, and my questions have been answered satisfactorily.

2. I understand that my participation is voluntary and that I am free to withdraw at any time

without giving any reason and without my medical care or legal rights being affected.

3. I understand that relevant sections of my medical notes and data collected during the

study may be looked at by individuals from Cardiff University, from regulatory

authorities or from the NHS Trust, when it is relevant to my taking part in this research.

I give permission for these individuals to have access to my records.

4. I agree to my GP being informed of my participation.

5. I agree that the clinicians involved in my primary care will be informed about

any psychiatric issues that are discovered by the questionnaires/interviews used

in the study and that are not covered by my current treatment.

6. I agree to take part in the above study.

_______________________________________________________________________________

Name of Participant Date Signature

______________________________________________________________________________

Name of Person Date Signature

Taking Consent

When completed: one copy for participant; one copy for researcher site file
