# Supplementary material for: Neurofeedback training for alcohol dependence versus treatment as usual: study protocol for a randomized controlled trial
Source: Trials. 2016 Oct 3;17:480. doi: 10.1186/s13063-016-1607-7 (PMC5048603; doi:10.1186/s13063-016-1607-7)
Supplement: Additional file 3: — Consent Form (MRI training). (DOCX 132 kb) [file 13063_2016_1607_MOESM3_ESM.docx]

CUBRIC, SCHOOL OF PSYCHOLOGY, CARDIFF **UNIVERSITY – MRI UNIT CONSENT FORM**

**Neurofeedback & Alcohol Dependence (Training Group), Version 1, 1 October 2014**

NAME OF PARTICIPANT ……………………… Sex: M / F Date of Birth:………………………

Please read the Volunteer Information Sheet and then read the following statements carefully and then add your signature. If you have any questions, please ask the person who gave you this form. You are under no pressure to give your consent and you are free to withdraw from the MRI examination at any time. By signing the form you are agreeing to the following:

- I understand that I am to take part in a functional MRI experiment in which I will be placed in the scanning machine for up to an hour, while my brain activity will be measured by the machine. During the scan I will view alcohol-related and other pictures and do neurofeedback training.
- I confirm that I have read and understand the MRI Volunteer Information Sheet and have had the opportunity to ask questions about it.
- I understand that upon completion of the study, I will receive £10/hour for my participation.
- I understand that participation in this study is entirely voluntary and that I can withdraw from the study at any time without giving a reason and without loss of credit / payment (as applicable).
- I understand that I am free to ask any questions at any time and that I am free to withdraw or discuss my concerns with the lead researcher (Prof. David Linden) or the research team (Dr Leena Subramanian, Dr Joseph Whittaker).
- I also understand that at the end of the study I will be provided with additional information and feedback about the purpose of the study.
- I understand that I can talk to the operators via an intercom and that I will be given an alarm “squeeze ball” that I can squeeze at any time to end the scan and signal to the operator.
- I understand that I can require, for any reason and at any time that I be immediately removed from the MRI machine.
- I understand and agree that the MRI scan is not a medical screening procedure and that the researchers are not qualified to provide a clinical diagnosis or identify potential abnormalities. However, if the researchers are concerned that there may be a potential abnormality on the scan, I consent to their disclosing the scan to a radiology consultant to provide a radiological report on the scan. I further consent to the results of this report being disclosed to my General Practitioner, if appropriate.
- I have completed the initial screening form and have been told that it is safe for me to be scanned.
- I understand that the information provided by me will be held confidentially, such that only the researchers can trace this information back to me individually. The information will be retained for up to 15 years after which it will be deleted/destroyed. I understand that I can ask for the information I provide to be deleted/destroyed at any time.

I, ___________________________________(NAME) consent to participate in the study conducted by School of Psychology, Cardiff University.

Signed:

Date:

*Do not write beneath this line, For Staff Use Only*

CUBRIC UNIQUE IDENTIFIER:………………………………

Statement by the Researcher carrying out the scan:

I certify that the above participant signed this form in my presence. I am satisfied that the participant fully understands the statements made and I certify that he/she had adequate opportunity to ask questions about the procedure before signing.

Signature………………………… Name……………………… Date ……………
